# President's Address—Florida State Dental Society

**Published:** 1907-12

**Authors:** 


### PRESIDENT'S ADDRESS—FLORIDA STATE DENTAL SOCIETY.

Once again we have assembled at this beautiful and pleasant seaside resort in annual session of the Florida State Dental Society, and as it is expected, and in fact required by the by-laws, that the president deliver an address at its annual meeting, I therefore, in the first place, wish to give you a cordial and hearty welcome to this, the twenty-fourth annual meeting of the Florida State Dental Society.

I hope that each one of you have come to this meeting with a full determination to do whatever you can to make it a successful one, and that each of you will get all the good you can from it, so that when we have adjourned to meet again a year hence, we may all say with earnestness that the meeting has been a successful, profitable and pleasant one.

For the confidence you have reposed in me at various times, and for the honor you conferred on me at the last meeting, by electing me as president of the society for the past year, and to preside over the deliberations of this body at this meeting, I desire to say that words are inadequate for me to express my appreciation.

In presiding at this meeting I shall endeavor to carry out the laws of the society, and to render any and all decisions impartially and in fairness to all. I trust, however, that all our deliberations and discussions may be of such character that there will arise no frictions between members, and no unpleasant personalities indulged in by any one, which will serve to mar the meeting in any way.

As professional men, working in a common cause, we should be considerate of each other's welfare, and do and say only those things which will have a tendency to elevate the standard of our profession in the eyes of the public.

Dr. Frank Holland of Atlanta, while president of the Georgia State Dental Society, in making his address before the society, said: "It shall be my honest endeavor to be fair and just to all; at the same time you must realize that unless the rules are enforced, the measure of success and dignity which we all desire, cannot be attained. I beg that you be patient and tolerant, and that your criticisms be tempered with mercy, allowing me credit for honesty of purpose and a strong desire for the welfare of our organization." I desire that you accept the sentiment therein expressed as applicable to my position at this time, and if perchance I should make a mistake, it will be one of the head and not of the heart, or at least not intentional.

Twenty-three years ago a few of us met in the city of Jacksonville and organized this dental society. Some of those who were at that first meeting have been called hence and gone to "that bourne from which no traveler ever returns," and some have retired from the activities of the profession; yet the society has gone on from year to year, doing much good for the profession and the public, and for many of us individually.

The dental society has a great work to do in the elevation of the standard of the dentists of the state, and in bringing about a fellowship between the dentists, which can only be accomplished by close association and contact with each other, in its pleasant and profitable sessions. The organization and continuance of dental societies by the various states and the enactment and enforcement of proper laws governing the practice of dentistry, has done as much toward the elevation of the standard of our profession as the efficient attainments of the individual. They have had a large share in bringing about our present state of advancement, and will continue to do for every dentist, who associates himself with the organization, and will attend its meetings, what nothing else will do for him. No member can stay at home and read all of the proceedings of the session, and get the good that he might derive from an attendance of the meeting. The society inculcates the spirit of ethics among the great body of practitioners, and serves to keep them out of ruts, and imparts to them advanced ideas of a professional nature which may not be attained in any other way. Every progressive and ethical member of the profession should be a member of the state dental society, and in fact I might say every one—progressive or non-progressive—should associate himself with others in society work, and by such association and exchange of ideas and methods of practice, be of mutual benefit to each other. The progressive will be stirred to greater progressiveness, and the non-progressive one will certainly imbibe from others a spirit of improvement, which may develop him into a progressive dentist, and in future be a credit to himself, to his society and to his community.

There are undoubtedly many dentists in this state, who are not yet members of this society, and who possibly, in the narrowness of their minds, think that associating themselves with others in an organization of this kind will do them no good. How great a mistake does one make who neglects to associate himself with his fellow practitioners in society work! It matters not how well educated he may be, a failure on his part to co-operate with others in up-building the profession at large, and educating the public to recognize and patronize the ethical, law-abiding and progressive dentist, is a failure to do a duty which will, like a boomerang, return and strike him with a force which will be felt for many years.

Every ethical dentist in this state, should therefore be a member of this society, and every effort should be made by those who are now members, to induce and secure the membership of all worthy ones, and thus increase the usefulness of the society to the greatest number of deserving dentists. I am pleased to note that for the past several months our corresponding secretary has been sending out to every member of the society, and to others who are not members, a blank application for membership, requesting that it be filled out and presented at this meeting.

What has been the result? How many new members will the society get through the efforts of the corresponding sectary? What have you done to aid in this increase of membership?

Membership in this society should be of more value to the dentist than simply entitling him to attend its sessions and participate therein. A dentist values his credentials from his alma mater, and points with pride to his diploma, hanging on the wall of his office, which certifies that he has completed the prescribed course, and is entitled to practice the art and science of dentistry.

So also should a dentist be proud of his membership in the Florida State Dental Society, and be able to point to a certificate of membership which certifies that he is an ethical dentist, abiding by the laws of the society, of the state, and of his community, and thus entitled to the patronage of the public.

The public should, therefore, be educated up to and instructed in the fact that such who possess this certificate of membership are more deserving of their patronage than the indifferent one who is not a member of the society, and does not display such certificate.

I would therefore suggest that a certificate of membership be devised and lithographed, and furnished to every member of this society who is in good standing. Let it be understood that such certificate shall be nicely framed and displayed in the office where it may be recognized by the public. Let it also be understood that if at any time the holder thereof should for any cause be suspended or dropped from membership in this society, the president or corresponding secretary shall call in the certificate, and the holder thereof shall surrender the same, or put himself or herself in such relation to the society that he or she may retain it.

Occasionally some member of the society will violate the code of ethics, who if admonished and counseled with in the proper spirit may be reclaimed and continued in the proper relation to the society and the profession. I would therefore suggest that a committee of ethics be appointed, whose duty it shall be to take cognizance of all violations of the code of ethics by members of the society, and admonish them and endeavor to cause them to refrain from such, and reclaim them.

If however, the offenders, after being admonished and counseled with, continue such violations, the committee shall report them so the Executive Committee and to the society at its next annual meeting, when such action shall be taken against them for suspension or expulsion.

Last year it was decided that this meeting should be one of practical value to those attending, by having a number of clinics. I am pleased to note by the program that the committee has made ample provision along that line, and it is hoped that each and all of you will at least learn some new thing, get some new idea, or some practical point which will be of value to you when you return to your office.

And now since there is always more or less recreation and pleasure looked forward to at these meetings, I would bid you have as good a time as possible in any legitimate way, and if you are disposed to disport yourselves in the waters of the broad Atlantic Ocean, you have every opportunity afforded you by this magnificent hotel, with its well equipped seaside bath rooms.

